# Experiences of a virtual day program for adolescents with eating disorders: a qualitative analysis of benefits and barriers

**DOI:** 10.1186/s40337-023-00859-z

**Published:** 2023-08-10

**Authors:** Vanessa Catenacci, Jennifer Couturier

**Affiliations:** 1 DeGroote School of Medicine, McMaster University, Hamilton, ON Canada; 2 Department of Psychiatry and Behavioral Neurosciences, McMaster University, Hamilton, ON Canada

**Keywords:** Eating disorder, Adolescent, Virtual day treatment, Qualitative

## Abstract

**Background:**

Throughout the COVID-19 pandemic, there was a detrimental impact to the symptoms and treatment of eating disorders, causing an increase in medical admissions and visits. Day treatment programs (DTPs), often used to bridge the gap between inpatient and outpatient treatment, were converted to online formats. This study aims to explore the impact of the transition to virtual DTPs on eating disorder treatment from the perspective of adolescents, their caregivers, and program staff.

**Methods:**

Twelve participants (3 adolescents, 4 caregivers, 5 healthcare providers) in a virtual day treatment program were interviewed using a semi-structured interview guide. Interviews were transcribed and managed with qualitative software NVivo 11.0. Conventional analysis was used to inductively identify pertinent themes related to patient, caregiver, and healthcare staff perceptions and experiences of the virtual day treatment. Summative content analysis provided counts of the barriers and benefits of virtual day treatment as identified by participants.

**Results:**

The majority of participants (10/12) had exposure to both virtual and in person settings, most participants (11/12) felt in-person day programs would be superior to virtual programs. Common limitations of the virtual format were feelings of isolation, less support from healthcare providers, parental burnout, and increased disordered eating. Common benefits were increased accessibility, parental involvement, improved communication with healthcare staff, and the ability for participants to be in their home environment and eat home food. Suggestions for improvement included designing a hybrid model of day treatment, increased family involvement, extending the day program to include dinners with family, and screening for patient appropriateness for the virtual setting.

**Conclusion:**

This qualitative study suggests that there are many barriers to effective implementation of virtual day programs. However, the virtual DTP program offers increased accessibility to patients during a period of a health pandemic and to patients in rural/remote areas with limited treatment options. Suggestions provided by participants in this study, such as increased family involvement, frequency of in person check-ins and increased number of meals supported by the program, may help to improve outcomes in virtual day treatment programs.

**Supplementary Information:**

The online version contains supplementary material available at 10.1186/s40337-023-00859-z.

## Abstract (plain language summary)

Eating disorders are serious psychiatric conditions that are detrimental to many adolescents and their families. The COVID-19 pandemic exacerbated the impact of eating disorders on adolescents due to social isolation, and increased levels of anxiety and depression. In addition, many of the treatment programs were converted to an online format. Day treatment programs, which normally involve patients spending the day at the hospital and returning home for the evening with their families, were converted to an online format. Although there is a lot of evidence supporting in-person day programs, there is limited research on the perspectives of various stakeholders on virtual programs. The goal of this study was to interview adolescents, caregivers and healthcare staff that had participated in a virtual day treatment program during the pandemic to understand their perspective on the pros and cons of the program. The results of this study and interviewee recommendations for program improvement are especially important to help patients who cannot access in-person care during health pandemics, those in rural and remote communities, and to provide support to those awaiting in-person care.

**Supplementary Information:**

The online version contains supplementary material available at 10.1186/s40337-023-00859-z.

## Introduction

Eating disorders (ED) are serious psychiatric conditions affecting up to 4% of adolescents and are highly debilitating for patients and families [1]. Prior to the COVID-19 pandemic, research demonstrated that 30–50% of patients treated in an inpatient unit and discharged to outpatient care relapsed [2]. Day treatment programs (DTP) are an option to help bridge the gap between inpatient and outpatient ED treatment. They provide daily care while also allowing patients to maintain their social relationships, provide dietetic intervention, transfer skills learned in treatment to their home environment, and encourage more independence [3]. Previous studies have demonstrated that DTPs result in greater improvements in psychological symptoms relating to the Eating Disorder inventory, BMI, and depression and self-esteem when compared to outpatient treatments [4]. Other studies identified that these changes were maintained at 6 and 12-month follow-ups, supporting the effectiveness of DTPs [5]. Further long term outcomes related to BMI, relapse and readmission rates favor DTPs over in patient admission, endorsing the need for widespread use of these programs [5].

Throughout the unprecedented COVID-19 pandemic, there was a significant detrimental impact on individuals with eating disorders (ED). Higher levels of anxiety, stress, depression, and post-traumatic stress disorder symptoms were reported throughout the pandemic [6, 7]. For adolescents with eating disorders, evidence shows that medical admissions and emergency department visits related to eating disorders surged during the pandemic [8, 9]. As a result, the pandemic necessitated the creation of virtual DTPs. While utilized during the pandemic, these programs also offer the opportunity to increase access to eating disorder care for patients in rural/remote areas. However, while there is a growing body of evidence to support DTPs, there is limited research on the perspectives of those attending virtual programs. Currently, only four studies have reported outcomes related to a virtual DTPs, two exclusive to adolescents and two for adults [10–13]. Findings from two studies demonstrated that while patients felt they had increased flexibility and accessibility in these virtual treatment programs, there was anxiety around the impact of their treatment and loss of human connection [10, 11]. Further, several challenges were noted in these studies including adaptations to meal support and challenges facilitating groups virtually [10, 11]. One study compared outcomes in the virtual day program to a cohort who participated in the in-person program prior to the pandemic and found that outcomes were comparable [13]. A more recent study in November 2022 demonstrated that virtual and in-person programs had similar weight restoration outcomes and rates of medical, psychiatric, or residential treatment admissions during and after treatment [12]. However, acceptability ratings regarding the virtual interventions varied widely between studies, with one study suggesting youth preferred in person care while other studies demonstrated high satisfaction of care within adults and parents [10, 11]. None of these studies investigate whether acceptability ratings impact engagement and attendance with the virtual day program. Further, only one study evaluated long term outcomes regarding follow-up body weights and hospital readmissions [12]. Overall, only a limited number of studies on virtual day treatment programs exist, none of which qualitatively assessed data from all three key stakeholders: participants, caregivers and healthcare staff.

This study aimed to explore benefits, barriers, and suggestions for improvement of virtual delivery of care for the eating disorders DTP, thus, we were interested in participant’s perspectives as those in treatment, those supporting individuals in treatment, and those delivering the program. The themes identified through this study may contribute to the development of recommendations for the use of virtual day programs going forward. With COVID-19 variants continuing to emerge, it is possible that virtual care will be needed once again in the foreseeable future. At this time, no study has evaluated suggestions for improvement by the stakeholders themselves. Adaptations that focus on improving treatment suitability for key stakeholders can lead to improved engagement, acceptability, and clinical outcomes [14]. This is especially important to help patients who cannot access in-person care, such as those in rural and remote communities, and to provide support to those awaiting in-person care. In order to make these adaptations, a thorough understanding of patient, caregiver and healthcare provider perspectives is needed in order to tailor the programs to their needs.

## Methods

In accordance with the principles of qualitative description, this paper reports on the qualitative experiences of adolescents, caregivers and staff involved in a virtual day treatment program for adolescents with eating disorders at McMaster Children’s Hospital (MCH). We considered their experiences in relation to limited prior knowledge about delivery of virtual care for eating disorders. All participants were qualitatively and independently interviewed. Ethics approval was obtained from the Hamilton Integrated Research Ethics Board.

For this qualitative study, the principles of qualitative description were followed [15]. We completed semi-structured interviews with adolescents, caregivers, and healthcare providers who received or conducted virtual day treatment during the COVID-19 pandemic. Clinicians included child life specialists, nutritionists, medical practitioners and therapists.

### Treatment context and adaptations for online working

At McMaster Children’s Hospital, the Eating Disorder Program provides outpatient, day program, and inpatient services. The day program admits patients who have not responded to outpatient treatment. Prior to the COVID-19 pandemic, the day treatment program operated 5 days per week (Monday to Friday). Approximately 9 h of face-to-face contact was offered per day with a capacity for four families at any one time. Three meals (breakfast, lunch, and dinner) were offered throughout the day with the support of child life specialist, nutritionists, and social workers. Alongside meal support, other interventions included group therapy, dietetic reviews, medication reviews and weekly individual therapy. The multidisciplinary team comprised of psychiatry, paediatrics, psychology, nursing, child life specialists, and dieticians.

In response to government restrictions on face-to-face working due to the COVID-19 pandemic, the DTP was adapted for virtual care delivery. This included moving individual therapy, adolescent and parent groups, meal support and education support online using Zoom. Two virtually supervised meals (breakfast and lunch) were offered for five days a week (Monday to Friday), Additionally, there was a weekly in-person weight check in, completed at McMaster Children’s Hospital during an appointment with the nursing team. The online program ran for six weeks for each participant, with the option to extend six weeks. Four adolescents participated at a time. This timeline and number of participants was consistent with the in person DTP. Overall, the only changes to programming between the virtual and in person day programs was the removal of dinner in the virtual program, and time off camera/unsupervised to complete school work at home in the virtual program. There were two periods of the virtual DTP: April 2020 to September 2020, and January 2021 to March 2021.

### Setting and participants

The participants from the study were drawn from adolescents treated in the virtual DTP. Clinical criteria for inclusion in the DTP were: (a) 3 months of outpatient treatment without progress, (b) a diagnosis of any ED from a licensed psychologist, psychiatrist, or physician, and, (c) had a community team willing to follow them on discharge. The clinical exclusion criteria for admission to the DTP were any patient with active suicidal ideation or aggressive behaviour. The study inclusion criteria for adolescent participants were as follows (a) participation in virtual day treatment at McMaster, and (b) ages 10–17 at the time of treatment. The inclusion criteria for caregivers were (a) any individual who provided informal care or caregiving to an individual living with an eating disorder (e.g. parent, grandparent) that had attended McMaster’s virtual day treatment program. The inclusion criteria for staff was (a) those that had provided treatment to adolescents in our virtual DTP. Participants were required to speak and understand English. Any participants involved in the DTP from April 2020 to September 2020, and January 2021 to March 2021 were able to participate in the study.

### Participant recruitment

A total of ten adolescents participated in the virtual DTP during the period of interest. All ten of theses adolescents who had participated in the virtual DTP received an invitation to participate in the study, as did one of their parents who was listed as the primary contact. There were five frontline staff delivering the virtual program and all of them were invited to participate. To recruit adolescents, caregivers, and healthcare providers, an information flyer was provided to potential participants by email. All stakeholders received flyers via email for study recruitment at the time of HIREB ethics approval, in March 2022. Interested participants contacted the study’s contact email and additional information was provided. Interested participants were emailed a consent form over a confidential platform (SignNow) and a time was arranged for the interview process.

### Data collection

Data was collected through in-depth, semi structured interviews that lasted approximately 30–45 min. The semi-structured interview was developed by VC and JC and included general question’s about the participants experience with eating disorders, benefits and limitations of virtual and in person day treatment settings and suggestions for improvement of facilitation of the day treatment program. The full interview structure and timeline for interview completion can be found in Additional file 1: Interview guide. All video interviews were conducted on Zoom by VC, recorded, and transcribed to remove any identifying information. All participants were asked questions in relation to their experiences with the virtual day program, in-person program (if applicable), and recommendations for program improvement. All participant interviews were conducted within 18 months of their participation in the virtual day treatment program.

In addition to the interview, participants filled out a demographic survey on Qualtrics. This platform was chosen as it provided a quick, accessible and confidential method for participants to respond, either on their computer or mobile phone. Three surveys were created for adolescents, caregivers, or healthcare staff All participants were asked about their age, gender, ethnicity, religion and access to the internet. Adolescents were asked about their eating disorder diagnosis, and the year of their diagnosis. Caregivers were asked about their relationship to the youth in the virtual DTP, their education level and socioeconomic (SES) status. Healthcare staff members were asked their job title and the number of years they had worked in the eating disorder DTP.

### Data analysis

Conventional content analysis, a qualitative research method in which codes, categories, and themes from interview data, was used to identify pertinent themes related to patient, caregiver, and healthcare staff perceptions/experiences of virtual DTP [16]. Data was managed with the qualitative software NVivo 11.0. An initial screening of interview transcript data was done to create codes based on common themes. Codes were refined through re-readings of the transcript by two authors, any any text that did not fit an initial code was given a new code. Data that fit under each code was represented as a count in summative content analysis, chose to minimize subjective interpretation of the data [16]. Quotes within the manuscript were selected to contextualize these findings.

## Results

### Participant demographics

We recruited three adolescents, four caregivers, and five healthcare staff who either participated in or facilitated the virtual DTP (Table 1**).** Of the participants, 10/12 had participated in both the in-person and virtual DTP, and were able to provide insight into the strengths and weaknesses of each format.

**Table 1:** Demographic participants of study participants

	HCP	Caregiver	Adolescents
# of participants	5	4	3
Age	N/A	N/A	14 (67%), 16 33%)^1^
Gender (% female)	Cisgender female (100%)	Cisgender female (75%) Cisgender male (25%)	Cisgender female (67%), transgender female (33%)
Role	Child life specialist (40%), Dietician (40%), Child and Youth Worker (, 20%)	Father (25%), Mother (75%)	N/A
Ethnicity	European (80%), Other (20%)	European (75%), Other (25%)	European (67%), Other (33%)
Education level	N/A	Post-secondary (75%), High school (25%)	N/A
Household income	N/A	> $100,000 (100%)	N/A
Preference for in-person	5/5	4/4	2/3

^1^Ages of participants at the time of their involvement in the virtual day treatment program

### Qualitative results

For a summative description of the data, see Tables 2 and 3. As indicated above, perceptions of the virtual DTP was from a combination of health care providers (HCP), adolescents, and caregivers. Although the group generally reported more disadvantages than advantages to virtual care, it was evident that there is a place for virtual care and there were many suggestions on how to improve the program. Particularly, common themes for improvement focused on creation of a hybrid program, increased family involvement, and ensuring suitability for online programming were highlighted.

**Table 2:** Categories and subcategories emerging from qualitative analysis regarding benefits/limitations of virtual and in-person day treatment programs (12 participants)

Categories		Subcategories	Frequency
Pros of virtual		Accessibility	8 participants, 13 references
		Communication improvement between parents, patients and HCP	4 participants, 4 references
		Individualized food plans/preparation	7 participants, 10 references
		Home environment	3 participants, 10 references
		Increased parental involvement in treatment	2 participants, 4 references
		Parenting benefits (eg: reduced burnout, reduced commuting)	3 participants, 3 references
		Technology adaptations that allowed for new programming and 1:1 support	3 participants, 4 references
Cons of virtual		Increased disordered eating habits	8 participants, 11 references
		Increased isolation and decreased peer allyship	8 participants, 13 references
		Less support from HCP	8 participants, 17 references
		Parental burnout	4 participants, 10 references
		Internet access concerns	4 participants, 4 references
		Miscommunication between HCP and parents/patients	3 participants, 5 references
		Zoom fatigue	3 participants, 3 references
		Decreased participant engagement online	2 participants, 2 references
		Patient safety concerns	1 participant, 1 reference
Pros of In-person	Increased HCP support		9 participants, 14 references
	Consistent meal planning/avoidance of disordered eating behaviours		5 participants, 6 references
	Separation of home and treatment environment		3 participants, 5 references
	More hands-on therapeutic programming		5 participants, 5 references
	Increased social connection with other patients		2 participants, 3 references
	Improved HCP team communication		1 participant, 1 reference
	Program structure including dinner		1 participant, 1 reference
Cons of In-person	Decreased accessibility (increased travel time and cost)		6 participants, 9 references
	Isolation from social connections/friends		2 participants, 3 references
	Hospital food		1 participant, 1 reference
	HCP burnout		1 participant, 3 references
	Increased ED behaviours due to close proximity of patients		1 participant, 2 references

**Table 3:** Categories and subcategories emerging from qualitative analysis regarding suggestions for improvement for virtual day treatment programs (12 participants)

Categories	Subcategories	Frequency
Suggestions for improvement	Designing a hybrid model of day treatment	5 participants, 11 references
	More frequent in-person check-ins	5 participants, 6 references
	Increased support for parents	2 participants, 2 references
	Creation of satellite programs for day treatment	1 participant, 1 reference
	Managing rules and patient expectations ahead of time	2 participants, 2 references
	Screening patient appropriateness for the virtual setting	1 participant, 1 reference
	Extending virtual day program hours to include dinner	2 participants, 2 references
	Increased family involvement	8 participants, 21 references
	Increased HCP communication	2 participants, 2 references
	Ensuring stable internet access for participants	1 participant, 1 reference

#### Pros of virtual day treatment

Families found that the virtual aspect of the DTP made the program more accessible, and reduced the costs and challenges that came with travelling long distances and coordinating schedules. Adolescents also found that it allowed them to remain connected to their school and community. In particular, one adolescent stated:

“I think it was helpful that my parents didn't have to drive because we're an hour drive from McMaster…And there were some good things like I could do my school at the end of the day and visit people as well.” (Adolescent #1).

HCP noted that meal preparation in the adolescent’s home environment was a major advantage of the virtual DTP. It was reported that this allowed them to create meals similar to what they would be eating after the program, the food was more culturally appropriate, and it gave them practice eating in their home environment. One HCP stated:

“I would say the positives is that they are in their home environment so we can work with their whole environment to support them. They also have their home food that they are used to depending on their culture and ethnicity and they were able to bring those foods to their meals and snacks. It was definitely a positive that we could work with their home foods in their home environment and use their cultural background.” (HCP #4).

Finally, several individuals highlighted that communication actually improved between staff and caregivers in the virtual setting. Parents were often in the background during the virtual sessions, and would communicate through email at other times. In particular, one HCP stated:

“Yeah it's interesting because I don't feel like it's even that different from in-person. In some ways virtual communication was almost better because parents would often be kind of like somewhere in the background so we could like shout to them and say ‘hey XXXX is wondering about this’ or ‘hey can you make sure that you grab this” (HCP #2).

#### Cons of virtual day treatment

Although there were many positives noted about the virtual DTP, 11/12 participants had a strong preference for the in-person program. Several disadvantages to virtual delivery of day treatment were identified. Caregivers remarked about caregiver burnout, highlighting that the burden of responsibility fell on them to watch their child when they were off screen, and how behavior would rapidly change once they were no longer being watched on camera. They described feeling inadequately prepared and unsure of what their role in the day program was, and wished that they had more support/resources on how to address an adolescent with an eating disorder. One caregiver commented:

“In terms of major limitations is the behavior change and parent exhaustion. There is no rest with a child with an eating disorder and the exhaustion of trying to keep up a rigid schedule. it seemed like you were trying all these different things and it was blowing up in your face and there is no one to support you. You're dealing with screaming and kicking and punching and physical abuse and mental abuse from this child that you're trying to save and there is not one break. So the day hospital program in-person, if nothing else, gives parents time to gain their own sanity back for 12 weeks.”(Caregiver #2).

Several adolescents and HCP mentioned the increased disordered eating habits during online meals and the difficulty addressing it. HCP struggled with addressing disordered eating habits as they did not want to accuse adolescents and struggled to develop rapport in the online setting. Adolescents mentioned how it was much easier to cheat during meals, with some feeding their pets or replacing high calorie foods with lower calorie ones. One adolescent stated:

“You could say ‘Oh yeah I had all this’ and kind of trick the nutritionist. You could also say that you're doing great when in reality you are not because it just feels much easier to kind of lie over the screen. A big part of eating disorders is that it is super manipulative so it will make you do that, so yeah it's kind of hard. When you are given the chance you probably will lie or not tell the truth.”(Adolescent #1).

Another concern from all parties was feelings of isolation and decreased peer allyship in the online setting. Adolescents commented that they felt isolated from both their friends at school, and the other patients in the program. HCP noted that it was difficult to engage patients online, build rapport, and have adolescents connect with one another. Parents also noted that adolescents were isolated in their rooms more, and seemed to be struggling to communicate their needs on camera. One parent stated:

“The downsides: I think there were a lot of isolation, sitting in your bedroom or some other closed off space in a home for 8 h a day I don't think is super healthy. I think it was bad enough during Covid that we had nowhere to go and nothing to do. Now we're further isolating. I know a lot of kids with eating disorders and other mental health issues, part of that is like ‘I'm going to stay in my bedroom and I'm not going to talk to my friends’. Well, does that feed that part of the eating disorder?”(Caregiver #1).

#### Pros of in-person day treatment

Of our 12 participants, 10 had experienced the in-person DTP. In general, the group reported more advantages rather than disadvantages to delivering eating disorder treatment in-person. HCP commented that there was increased ability to interpret body language, teach hands on skills, and supervise patients while in-person. Both adolescents that attended both in-person and virtual programs felt that communicating their needs and struggles was easier in-person. One adolescent stated:

“I would also say the ability to communicate face to face was a lot easier. I could reach out for help and pull someone aside and be like ‘hey I'm struggling with this’. Or like they could see it on your face, whereas over zoom it was more difficult.” (Adolescent #3).

As mentioned earlier, many parents described feelings of caregiver burnout in the virtual day program. Several parents felt the in-person program created a separation of home and treatment environment that provided both a reprieve for parents, and the opportunity for adolescents to develop more independence. One parent stated:

“She had become dependent on all the adults in her life to do things and be that emotional support. For us as caregivers that was super exhausting, because it was like having a newborn again. So I see that benefit of having her out of the home environment where all of this has happened, because I think there was a challenge to building independence when she was tied to what happened in the home.”(Caregiver #4).

#### Cons of in-person day treatment

While most people preferred in-person treatment to online, everyone acknowledged that accessibility was a large limitation for in-person, and virtual programs would ensure access to those in rural communities who could not travel. Further, several parents mentioned concern over incurred costs and time away from family as a result of the distance.

“It is a huge time commitment and cost, we are just over an hour away from Hamilton and driving there and back twice a day with current gas prices is a lot of commitment and disruption for the family. That meant leaving the house at 6:30 in the morning, missing getting my other children ready for school and out the door. I had to hire teenagers to come in to help with the other children and then was not able to return till 7:00 PM every night.” (Caregiver #2).

#### Suggestions for improvement

Although the majority of participants thought in-person treatment was better, they acknowledged the value of virtual programs and provided many suggestions on how the program should be improved. Eight participants highlighted the needed for increased family involvement and support. Several individuals highlighted that while there was some family involvement, some formalized teaching and instructions for parents would have been helpful. Further, educating siblings on what to expect at home and creating support groups for them was highlighted. One HCP stated:

“It would also be helpful to have the ability for parents to log in and learn. There are some parent groups that I know Mac offers but there are not really any sibling support groups which I think would be really beneficial because a lot of other illnesses offer sibling support groups for kids with disabilities or kids with cancer. It is not as easy to find something similar for kids with mental health issues or eating disorders specifically. I think it would be beneficial to offer some of that to the family and I think it would really nicely tie with the virtual program and being able to continue supporting the child through their meals after the in-person program was done.” (HCP #3).

Other participants suggested altering the structure of day treatment programs in a post-COVID era by creating a hybrid model of care. Several participants mentioned that in-person treatment was more valuable at the beginning of treatment, where more rigid observation was needed. However, they highlighted that virtual treatment was a valuable way to transition the individual back to their home environment, allowing them to eat their home foods and have dinner with their families while on Zoom. One parent stated:

“A hybrid model would be awesome where they do a few weeks of virtual and a few weeks in-person, but the in-person should be a good block of six weeks so that they have enough time to intensively and consistently see behaviors develop. Then they can go back to an at home environment to test the skills they learned.” (Caregiver #2).

Another parent stated that the main limitations to the virtual was the lack of support and peer connection, while the main limitation of in-person was accessibility. To address this, they suggested creating a satellite program as follows:

“That is a tricker to do, but if you create an environment that is virtual but reaching communities further away, maybe there is a way to bring participants together. So you have 2–3 patients in one location in-person, but on camera with the main team at McMaster. Maybe you have a dietician physically with those kids at the satellite space so there is a professional on site. So it is like a satellite program where they can patch into the main program going on somewhere else. That may not be feasible but having that professional in-person would make a big difference.” (Caregiver #3).

## Discussion

To our knowledge, this is the first study to qualitatively evaluate the perspective for pediatric populations during the COVID-19 pandemic using an interview format. Experiences of virtual day program implementation were captured through structured interviews consisting of child life specialists, nutritionists, caregivers, and adolescents. Based on our qualitative findings, virtual day treatment is acceptable and feasible among teams delivering and families receiving treatment. However, there were some significant limitations that could be addressed by the suggested improvements in this study. There was also a clear preference from families and HCPs for in-person or hybrid models of treatment.

With respect to its advantages, interviewees commented on the increased accessibility, benefits of food preparation at home, and parental involvement of virtual day program. Interviewees recognized decreased HCP support, caregiver burnout, and increased disordered eating behaviors as limitations to the virtual setting. Adolescents described feelings of isolation and less peer allyship due to an inability to connect with others over Zoom, while HCP stated that difficulties in rapport-building made it more challenging to develop a therapeutic alliance. While recalling meal preparation, all parties felt that meals with home foods created a more realistic setting for treatment. Overall, almost all parties agreed that while in-person treatment was superior, virtual programs offered an opportunity to provide increased access to treatment for individuals with limited options.

Similar to our results, a recent mixed methods study on delivering virtual care for mental health during the pandemic found that mental health clinicians thought virtual platforms were easy to operate and improved access to care for their patients and families [17]. Conversely, the same study highlighted technical difficulties and trouble managing disruptions in their patients’ homes as strong challenges [17]. Overall, the clinicians felt that virtual care impacted their patient interactions, particularly when it came to rapport building and managing patient privacy in their homes. Surprisingly, patient privacy concerns were not mentioned by any of the participants in our study, with many suggesting that being in their home environment with their home cuisine was actually a positive aspect of the virtual day program. Reduced HCP support and rapport building was identified as a disadvantage by the majority of participants in our study. Another study found that adolescents and parents felt that while virtual day treatment was helpful and accessible, it was less comfortable than in-person treatment and did not provide as strong a connection with healthcare staff. This suggests a need to find new ways of building trusting relationships and a deeper connection online if virtual programs are to be used beyond the pandemic [18]. Further, another case study involving three young women with eating disorders who received a virtual, home-based treatment model of care found perceptions of virtual treatment similar to our study [19].

In regards to the safety of online treatment methods, a recent study in the UK determined that it was relatively safe to deliver day treatment on the virtual platform with no serious incidents recorded in that time period, with weekly check-ins being vital to managing risks to health [20]. However, other studies pointed out that adapting DTP during the pandemic presented the largest challenge of all treatment programs, as it caused a reactivation of ED related symptoms, self-injurious behaviors, and suicide risk [21]. Within our study, there was no anecdotal evidence of safety concerns, however several participants referenced increases in disorder eating. In 2021, Canadian Practice Guidelines were published to evaluate the evidence on virtual care focused specifically on children and adolescents (< 18 years) and emerging adults (18–25 years) with EDs, in the COVID-19 context. While recommendations were provided surrounding family-based therapy, cognitive behaviour therapy, and in-person medical evaluations, there was no recommendations on the effectiveness or safety of more intensive services, such as virtual day hospitals as there was no evidence to draw upon [22]. Therefore, more research and standardized protocols are needed to ensure safety before widespread implementation of virtual day programs.

Overall, our results suggest the importance of testing hybrid models of DTP, as multiple participants expressed an interest in day programs that contain in-person and virtual components. Alternatively, other participants suggested satellite programs where 2–3 patients from the same region would be supervised in-person by an HCP from that region and zoom into the main campus. While this presents many logistical challenges, it would allow for increased accessibility and ability to care for more patients, while still maintaining an in-person connection. Similar suggestions have been seen in previous studies. Notably, parents interviewed in a 2022 study on virtual family-based treatment for adolescent anorexia strongly suggested a hybrid model of treatment that would allow for convenience while still maintaining therapeutic rapport [23]. Alternatively, other participants proposed other suggestions for improved virtual day treatment. This included increased support for parents, screening patient appropriateness, extending day program to include dinners, and increased family involvement in treatment.

There are several limitations to our study. Primarily, our sample size was small, impacting whether the themes discussed in our study would be representative to a larger population. Our research team made several attempts to encourage all those who had participated to enroll in the study, with several consented participants lost to follow up. Further, the demographics of individuals indicates that our population was predominantly European and female, and may not accurately reflect all populations. In addition, an advantage of our study was that 11/12 participants engaged in both the in-person and virtual day setting, and were able to make direct comparisons between the two settings. However, the in-person experiences were a mix of both during COVID-19 requirements (eg: masking) and before the pandemic, which may have resulted in significantly different experiences for individuals. Finally, while the pandemic provided an opportunity to explore virtual treatment methods, the scenario created in this study may not be more largely applicable to experiences of rural and remote patients/stakeholders regarding isolation and accessing services. For example, this virtual program still had an in-person weight component, which may be more difficult for rural/remote participants. Further, the pandemic created an environment where feelings of isolation perpetuated all aspects of lives, not just the ED day treatment program. In comparison, participants in rural/remote locations still have access to community (ie: friends/family) support.

Overall, this study supports the need for further examination of the utility of virtual day treatment programs. As new COVID-19 variants emerge and potential ‘waves’ create uncertainty, it is safe to assume that at one point day treatment programs may return to virtual settings. Further, the shift to virtual care in the general medical field may cause patients to opt for virtual rather than in-person care given the increase in accessibility and reduction in cost [24]. Finally, the creation of strong virtual programs offers increased accessibility to patients who cannot access in-person care, such as those in rural and remote communities, and to provide support to those awaiting in-person care. Going forward, there are several considerations when implementing a virtual day treatment format based on our participant responses. First, increased family involvement and caregiver support, such as formalized teaching/health education sessions for family members, or the formation of caregiver support groups, could possibly help combat caregiver burnout and clarify their role in the process. Next, screening patient appropriateness for the virtual day setting based on co-morbid medical conditions, severity of illness, and agreeableness to treatment. Third, including dinners on the program in a manner similar to that of the in person day treatment structure to maximize treatment exposure. Finally, as suggested by several participants, we should consider alternative models of care such as hybrid programs or satellite in person programs and compare these to our current in person and virtual models.

### Supplementary Information

**Additional file 1**. Interview guide.

## Data Availability

Data will be shared on request.

## Abbreviations

DTP: Day treatment program
ED: Eating disorder
HCP: Healthcare provider
MCH: McMaster Children’s Hospital
SES: Socioeconomic status

## References

[1] Hoek HW, van Hoeken D (2003). Review of the prevalence and incidence of eating disorders. Int J Eat Disord.

[2] Lock J, Le Grange D. Family‐based treatment: where are we and where should we be going to improve recovery in child and adolescent eating disorders. Int J Eat Disord. 2019;52:481-487. 10.1002/eat.22980 30520532

[3] Zipfel S, Reas DL, Thornton C, Olmsted MP, Williamson DA, Gerlinghoff M (2002). Day hospitalization programs for eating disorders: a systematic review of the literature. Int J Eat Disord.

[4] Kong S (2005). Day treatment programme for patients with eating disorders: Randomized controlled trial. J Adv Nurs.

[5] Hepburn ZM, Rothwell ER (2020). Effectiveness of day treatment for eating disorders: are improvements maintained at 12-month follow-up?. Ment Health Rev J.

[6] Devoe D, Han A, Anderson A, Katzman DK, Patten SB, Soumbasis A (2023). The impact of the covid -19 pandemic on eating disorders: a systematic review. Int J Eat Disord.

[7] Colleluori G, Goria I, Zillanti C, Marucci S, Dalla RL (2021). Eating disorders during COVID-19 pandemic: the experience of Italian healthcare providers. Eat Weight Disord.

[8] Toulany A, Kurdyak P, Guttmann A, Stukel TA, Fu L, Strauss R (2022). Acute care visits for eating disorders among children and adolescents after the onset of the COVID-19 pandemic. J Adolesc Health.

[9] Agostino H, Burstein B, Moubayed D, Taddeo D, Grady R, Vyver E (2021). Trends in the incidence of new-onset Anorexia Nervosa and atypical Anorexia Nervosa among youth during the COVID-19 pandemic in Canada. JAMA Netw Open.

[10] Plumley S, Kristensen A, Jenkins PE (2021). Continuation of an eating disorders day programme during the COVID-19 pandemic. J Eat Disord.

[11] Brothwood PL, Baudinet J, Stewart CS, Simic M (2021). Moving online: young people and parents’ experiences of Adolescent eating disorder day programme treatment during the COVID-19 pandemic. J Eat Disord.

[12] Van Huysse JL, Prohaska N, Miller C, Jary J, Sturza J, Etsell K (2022). Adolescent eating disorder treatment outcomes of an in-person partial hospital program versus a virtual intensive outpatient program. Int J Eat Disord.

[13] Blalock DV, Le Grange D, Johnson C, Duffy A, Manwaring J, Tallent CN (2020). Pilot assessment of a virtual intensive outpatient program for adults with eating disorders. Eur Eat Disord Rev.

[14] Rooijen M, Lenzen S, Dalemans R, Beurskens A, Moser A (2020). Stakeholder engagement from problem analysis to implementation strategies for a patient-reported experience measure in disability care: a qualitative study on the process and experiences. Health Expect.

[15] Sandelowski M (2000). Whatever happened to qualitative description?. Res Nurs Health.

[16] Hsieh HF, Shannon SE (2005). Three approaches to qualitative content analysis. Qual Health Res.

[17] Romanchych E, Desai R, Bartha C, Carson N, Korenblum M, Monga S (2021). Healthcare providers' perceptions of virtual-care with children's mental health in a pandemic: a hospital and community perspective. Early Interv Psychiatry.

[18] Stewart C, Konstantellou A, Kassamali F, McLaughlin N, Cutinha D, Bryant-Waugh R (2021). Is this the ‘new normal’? A mixed method investigation of young person, parent and clinician experience of online eating disorder treatment during the COVID-19 pandemic. J Eat Disord.

[19] Latzer Y, Herman E, Ashkenazi R, Atias O, Laufer S, Biran Ovadia A (2021). Virtual online home-based treatment during the COVID-19 pandemic for ultra-Orthodox young women with eating disorders. Front Psychiatry..

[20] Obiekezie A, Friel C, Pathan MT (2022). Safety of delivering eating disorders day treatment programme on the virtual platform in (COVID-19) pandemic. BJPsych Open.

[21] Graell M, Morón-Nozaleda MG, Camarneiro R, Villaseñor Á, Yáñez S, Muñoz R (2020). Children and adolescents with eating disorders during COVID-19 confinement: difficulties and future challenges. Eur Eat Disord Rev.

[22] Couturier J, Pellegrini D, Miller C, Bhatnagar N, Boachie A, Bourret K (2021). The COVID-19 pandemic and eating disorders in children, adolescents, and emerging adults: virtual care recommendations from the Canadian consensus panel during COVID-19 and beyond. J Eat Disord.

[23] Couturier J, Pellegrini D, Grennan L, Nicula M, Miller C, Agar P (2022). A qualitative evaluation of team and family perceptions of family-based treatment delivered by videoconferencing (FBT-V) for adolescent anorexia nervosa during the COVID-19 pandemic. J Eat Disord.

[24] Rivett M (2020). Relational lockdown and relational Trauma(dagger) in the time of coronavirus: a reflection from a UK family therapist. Fam Process.

