# Supplementary material for: Experiences of a virtual day program for adolescents with eating disorders: a qualitative analysis of benefits and barriers
Source: J Eat Disord. 2023 Aug 10;11:133. doi: 10.1186/s40337-023-00859-z (PMC10413601; doi:10.1186/s40337-023-00859-z)
Supplement: Supplementary file 1 — Additional file 1. Interview guide. [file 40337_2023_859_MOESM1_ESM.docx]

**Additional File 1: Interview guide.**

**Interview guide for adolescents, caregivers, and healthcare staff**

**notes in italics are intended for the study interviewer; these comments are not to be read*

*aloud to the study participant.**

**Interview #1: Youth (12-17) participating in a virtual day program**

**Introduction**

Thank you for agreeing to participate in this interview today. You were invited to talk today because you currently are or have been a participant in a virtual eating disorder program at McMaster. The purpose of this interview is to learn about your experiences and perspectives on the delivery of virtual day programs for adolescent eating disorder treatment.

**General Introduction About the Interviewee’s Experiences:**

1. Can you tell me about your eating disorder? How long have you been diagnosed?
2. What treatments have you had in the past?
3. How long did you participate in the virtual day program for?

**Identification of the Problem**

*The intent of this series of questions is to understand the patients experiences with virtual day programs for eating disorder treatment. What were their experiences and challenges?*

1. Can you tell us about your experience with virtual adolescent eating disorder day programs? What does a typical week in the day program look like to you?
2. What are some of the positives/benefits about the virtual day program?
3. From your experience, what are some barriers or limitations you’ve seen in the delivery of virtual day programs?
4. How do you feel virtual day programs have impacted your overall quality of treatment? What do you find the most and least helpful parts of virtual day programs?

**Comparison of Virtual vs. In-Person Day Programs:**

*This section is only applicable if the individual participated in an In-Person Day Program prior to the pandemic. The research team is interested in understanding how in-person and virtual day program experiences compare, with the goal of identifying the benefits and limitations of each treatment delivery style.*

1. Can you tell us about your experience with in-person day programs?
2. What were some of the advantages of virtual day programs compared to in person?
3. What were some of the limitations of virtual day programs compared to in person?
4. Going forward, which program style would you prefer?

**Improving Facilitation of Virtual Day Programs:**

*In this series of questions, the research team is interested in strategies to improve virtual delivery of day programs.*

1. How do you think social support systems (ie: your parents, caregivers, or friends) can aid in the effectiveness of virtual day program delivery?
2. Do you have any thoughts on how virtual day programs can be improved?

**Healthcare Team Role in Virtual Program Delivery:**

*The intent of this series of questions is to understand participant perspectives of how healthcare teams can improve support of patients and families in a virtual setting.*

1. How do you think communication between healthcare teams and patients/caregivers has been impacted in virtual settings?
2. Do you have any thoughts on how these barriers could be overcome?
3. How would you like to be supported by healthcare teams?

**Interview #2: Caregivers**

**Introduction**

Thank you for agreeing to participate in this interview today. You were invited to talk today because you are currently a caregiver to an adolescent who is participating or has participated in a virtual eating disorder program at McMaster. The purpose of this interview is to learn about your experiences and perspectives on the delivery of virtual day programs for adolescent eating disorder treatment.

**General Introduction About the Interviewee’s Experiences:**

1. Can you tell me about your child’s eating disorder? How long have they been diagnosed and/or undergoing treatment?
2. What treatments have they had in the past?
3. How long did they participate in the virtual day program for?

**Identification of the Problem**

*The intent of this series of questions is to understand the patients experiences with virtual day programs for eating disorder treatment. What were their experiences and challenges?*

1. What did your child’s typical week in the virtual day program look like to you?
2. Can you tell us about your experience with virtual adolescent eating disorder day programs? (ie: what was your role as caregiver to someone participating in these programs)
3. What are some of the positives/benefits about the virtual day program?
4. From your experience, what are some barriers or limitations you’ve seen in the delivery of virtual day programs?
5. How do you feel virtual day programs have impacted your child’s overall quality of treatment?

**Comparison of Virtual vs. In-Person Day Programs:**

*This section is only applicable if the individual participated in an In-Person Day Program prior to the pandemic. The research team is interested in understanding how in-person and virtual day program experiences compare, with the goal of identifying the benefits and limitations of each treatment delivery style.*

1. What did your child’s typical week in the in-person day program look like to you?
2. Can you tell us about your experience with in-person day programs? ((ie: what was your role as caregiver to someone participating in these programs)
3. What were some of the advantages of virtual day programs compared to in person?
4. What were some of the limitations of virtual day programs compared to in person?
5. Going forward, which program style would you prefer?

**Improving Facilitation of Virtual Day Programs:**

*In this series of questions, the research team is interested in strategies to improve virtual delivery of day programs.*

1. How do you think social support systems can aid in the effectiveness of virtual day program delivery?
2. Do you have any thoughts on how virtual day programs can be improved?

**Healthcare Team Role in Virtual Program Delivery:**

*The intent of this series of questions is to understand participant perspectives of how healthcare teams can improve support of patients and families in a virtual setting.*

1. How do you think communication between healthcare teams and patients/caregivers has been impacted in virtual settings?
2. Do you have any thoughts on how these barriers could be overcome?

**Interview #3: Healthcare Staff**

**Introduction**

Thank you for agreeing to participate in this interview today. You were invited to talk today because you are currently a healthcare team member delivering care/treatment at McMaster’s virtual eating disorder day program. The purpose of this interview is to learn about your experiences and perspectives on the delivery of virtual day programs for adolescent eating disorder treatment.

**General Introduction About the Interviewee’s Experiences:**

1. Can you tell me about your experiences working in delivering healthcare for eating disorder treatment?
2. How long have you been working at the virtual day program for?

**Identification of the Problem**

*The intent of this series of questions is to understand the patients experiences with virtual day programs for eating disorder treatment. What were their experiences and challenges?*

1. What does your typical week in the day program look like to you? Can you describe your role and your responsibilities?
2. What are some of the positives/benefits about the virtual day program?
3. From your experience, what are some barriers or limitations you’ve seen in the delivery of virtual day programs?
4. How do you feel virtual day programs have impacted your ability to provide counselling and/or treatment?

**Comparison of Virtual vs. In-Person Day Programs:**

*This section is only applicable if the individual participated in an In-Person Day Program prior to the pandemic. The research team is interested in understanding how in-person and virtual day program experiences compare, with the goal of identifying the benefits and limitations of each treatment delivery style.*

1. What did your typical week in the in person day program look like to you? Can you describe your role and your responsibilities?
2. What were some of the advantages/limitations of in person compared to virtual?
3. Going forward, what style of program (virtual or in person) do you believe would be more effective?

**Improving Facilitation of Virtual Day Programs:**

*In this series of questions, the research team is interested in strategies to improve virtual delivery of day programs.*

1. How do you think social support systems can aid in the effectiveness of virtual day program delivery?
2. Do you have any thoughts on how virtual day programs can be improved?

**Healthcare Team Role in Virtual Program Delivery:**

*The intent of this series of questions is to understand participant perspectives of how healthcare teams can improve support of patients and families in a virtual setting.*

1. How do you think communication between healthcare teams and patients/caregivers has been impacted in virtual settings?
2. Do you have any thoughts on how these barriers could be overcome?
